# Radiogenomics Reveals Correlation between Quantitative Texture Radiomic Features of Biparametric MRI and Hypoxia-Related Gene Expression in Men with Localised Prostate Cancer

**DOI:** 10.3390/jcm12072605

**Published:** 2023-03-30

**Authors:** Chidozie N. Ogbonnaya, Basim S. O. Alsaedi, Abeer J. Alhussaini, Robert Hislop, Norman Pratt, Ghulam Nabi

**Affiliations:** 1 Division of Imaging Science and Technology, University of Dundee, Dundee DD1 4HN, UK; 2 College of Basic Medical Sciences, Abia State University, Uturu 441103, Nigeria; 3 Statistics Department, University of Tabuk, Tabuk 47512, Saudi Arabia; 4 Department of Medical Imaging, Al-Amiri Hospital, Ministry of Health, Sulaibikhat 1300, Kuwait; 5 Cytogenetic, Human Genetics Unit, Ninewells Hospital and Medical School, Dundee DD1 9SY, UK; 6 School of Medicine, Ninewells Hospital, Dundee DD1 9SY, UK

**Keywords:** radiogenomics, bpMRI, prostate cancer, textural features

## Abstract

Objectives: To perform multiscale correlation analysis between quantitative texture feature phenotypes of pre-biopsy biparametric MRI (bpMRI) and targeted sequence-based RNA expression for hypoxia-related genes. Materials and Methods: Images from pre-biopsy 3T bpMRI scans in clinically localised PCa patients of various risk categories (n = 15) were used to extract textural features. The genomic landscape of hypoxia-related gene expression was obtained using post-radical prostatectomy tissue for targeted RNA expression profiling using the TempO-sequence method. The nonparametric Games Howell test was used to correlate the differential expression of the important hypoxia-related genes with 28 radiomic texture features. Then, cBioportal was accessed, and a gene-specific query was executed to extract the Oncoprint genomic output graph of the selected hypoxia-related genes from The Cancer Genome Atlas (TCGA). Based on each selected gene profile, correlation analysis using Pearson’s coefficients and survival analysis using Kaplan–Meier estimators were performed. Results: The quantitative bpMR imaging textural features, including the histogram and grey level co-occurrence matrix (GLCM), correlated with three hypoxia-related genes (ANGPTL4, VEGFA, and P4HA1) based on RNA sequencing using the TempO-Seq method. Further radiogenomic analysis, including data accessed from the cBioportal genomic database, confirmed that overexpressed hypoxia-related genes significantly correlated with a poor survival outcomes, with a median survival ratio of 81.11:133.00 months in those with and without alterations in genes, respectively. Conclusion: This study found that there is a correlation between the radiomic texture features extracted from bpMRI in localised prostate cancer and the hypoxia-related genes that are differentially expressed. The analysis of expression data based on cBioportal revealed that these hypoxia-related genes, which were the focus of the study, are linked to an unfavourable survival outcomes in prostate cancer patients.

## 1. Introduction

Several studies have suggested that respiration in cancer cells is impaired [1,2,3,4]. There is an observed increase in glycolytic rates of cancers cells even in normoxic conditions with cells shifting their metabolic needs towards oxidative phosphorylation [5,6]. The changed metabolic phenotype of cancer cells persists despite neovascularisation and is driven by genetic changes [7]. Moreover, hypoxia is established as a micro-environmental influencing factor in most solid tumours, leading to changes in transcriptomic profiles with potential metastasis and treatment resistance [8,9]. Hypoxia is a significant characteristic of prostate cancer [10], and it has been linked to poor prognostic indicators [11,12,13]. Whether recent advances in imaging, in particular MRI, could predict the expression of these genes with a degree of accuracy has not been explored in the past.

Radiogenomics research is growing to address the above question. The research entails a combination of large numbers of quantitative imaging features and gene signatures using computer algorithms [14]. This is a promising technology in cancer-related research and has been used in the prediction of tumour behaviour. It basically provides insight into the development, heterogeneity, and occurrence of tumours by deep mining the biological nature of medical images [15,16,17]. There are limited studies on radiogenomics in prostate cancer, and this is the first study to correlate bpMRI radiomic texture features using histogram and GLCM with hypoxia-related genes in prostate cancer. Histogram and GLCM characteristics are among the texture features [15]. GLCM measurements represent the contrast, energy, entropy, and homogeneity of cells and tissues and depict surface characteristics of cancers [18]. The smoothness or otherwise of GLCM is determined by the distribution of the grey level, and entropy and can be calculated from images [16,19,20]. There are reports of tumour heterogeneity correlating with GLCM [21,22]. It is also well-reported that hypoxia mediates tumour heterogeneity [23,24]. Various non-invasive established methods have been in use for the diagnosis of prostate cancer, which includes digital rectal exam (DRE) and prostate specific antigen (PSA). However, imaging has been considered a useful tool for risk-stratification and is the reason for the introduction of pre-biopsy MR imaging [17,25,26,27]. PSA estimation and various thresholds may be beneficial in the decision to perform a biopsy; in a recent study reporting on thresholds of pre-biopsy PSA for the prediction of advanced prostate cancer (defined as a Gleason score ≥ 7 at biopsy), a PSA ≤ 4.1 carried a negative predictive value of 95.1% [95% confidence interval (CI): 83.0–98.7] for men less than 65 years of age [28]. Callender et al. [29] have shown that a pre-biopsy MRI approach caused a 4.8-fold increase in MRI scans, but the overall cost was significantly lower than that with a biopsy-first approach. How the use of various prostate cancer biomarkers along with various PSA thresholds would improve the cost effectiveness of pre-biopsy MRI has not been shown.

We hypothesised that hypoxia-related genome expression in localised PCa correlates with tumour heterogeneity based on texture analysis and that this can be non-invasively detected using pre-biopsy bpMRI of the prostate. A hypoxic microenvironment and relatively hypoperfused areas in PCa would lead to changes in textural morphology and with the power of imaging data mining could form a radiogenomic signature of the disease.

The study aimed to report the correlation between radiomic texture features with hypoxia-related gene expression in clinically localised PCa. We envisaged that combining hypoxia-targeted genes with radiomic features extracted from bpMRI (T2WI & ADC) may help in developing imaging biomarkers for the risk stratification of prostate cancer for various therapeutic modalities of treatment.

## 2. Materials and Methods

**Figure 1:**
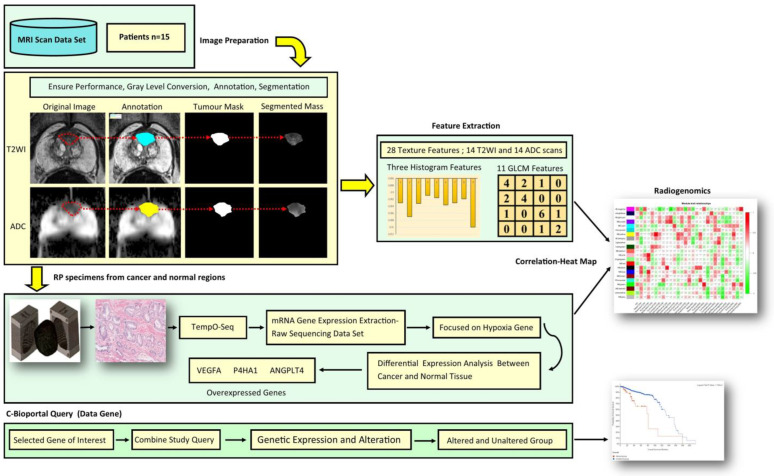
Radiogenomic study pipeline. Before extracting quantitative imaging texture features, the first stage shows a bpMRI segmented region of interest in both T2WI and ADC images. The second stage displays the use of the mould during prostate tissue slicing and histological preparation of tissue before hypoxia-related genes of interest were extracted using TempO-seq. Subsequently, differential expression analysis performed. The third step depicts the stage of exploratory analysis of the differentially expressed hypoxic genes of interest, which was performed based on cBioportal.

The study design and work flow are shown in Figure 1. Based on previous literature reviews, we focused on hypoxia-related genes (ANGPTL4, VEGFA, and P4HA1) [30,31,32,33]. A cross-sectional gene-oriented query on cBioprtal (https://www.cbioportal.org, accessed on 9 June 2021) (34) was conducted to validate the overexpressed hypoxic gene based on the database. cBioportal, a widely used web interface that gives access to public cancer genomics datasets, was utilised to retrieve the Cancer Genome Atlas data [34,35,36]. The focus gene-query for prostate cancer was chosen, with genomic information starting with HUGO genes. For the cBioportal pipeline, PCa studies filter the query by gene using HUGO gene symbols, sort tracks based on oncoprint, the cancer type summary, and plots, and mutations were investigated, including survival data. In 23 study reports of PCa based on cBioportal, Supplementary Figure S1 illustrates the gene query of overexpressed hypoxic genes.

### 2.1. Proof-of-Concept Cohort

**Figure 2:**
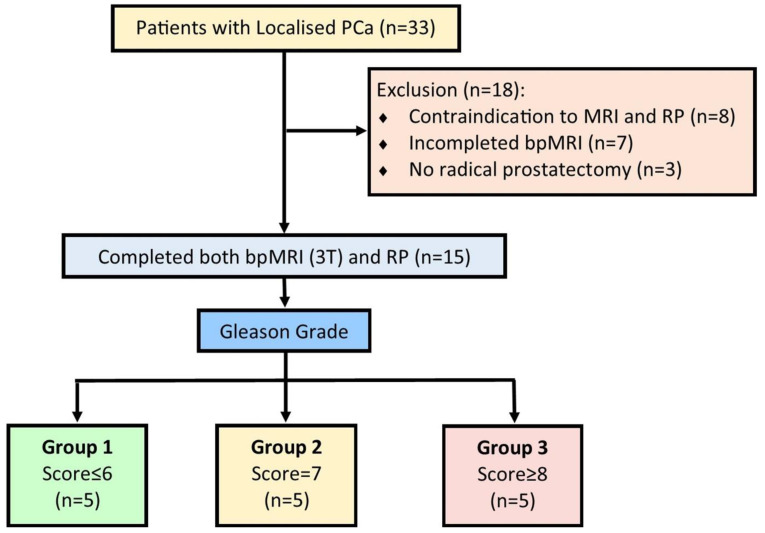
Exclusion and inclusion criteria for the prospective study.

This study was approved by the institutional research ethics committee (TR000532) and the Institutional Caldicott approval (IGTCAL no: 5816) for utilising radiological images. The study was conducted from February 2019 through December 2021. Thirty-three male patients with clinically localised PCa who were scheduled for radical prostatectomy were prospectively recruited. Fifteen patients were enrolled in this study after meeting the inclusion criteria of an abnormal digital rectal examination (DRE), PSA ≤ 20 ng/mL, clinically localised PCa with at least 10 years of life expectancy, prostate volume ≤ 80 mL, <T3 diseases, and capacity for informed consent. Exclusion criteria were the inability to give informed consent, locally advanced or metastatic disease, poor general health, a life expectancy of less than 10 years, or having been previously diagnosed with acute prostatitis within a year of undergoing radical surgery (Figure 2).

### 2.2. Biparametric MRI (BpMRI) Image Acquisition

Before radical prostatectomy, patients underwent bpMRI scans with a 3T scanner (TIM Trio, Siemens, Erlangen, Germany), focusing on T2WI and DWI. T2WI was acquired using a turbo spin-echo sequence with a resolution of about 0.5 mm in the plane with a slice thickness of 3.6 mm. DWI was acquired in a single-shot echo-planar imaging sequence with a resolution of 2 mm in-plane and 3.6 mm slice thickness with diffusion encoding gradients × 3 directions. The apparent diffusion coefficient (ADC) map was computed from DWI data (*b* values = 0, 400, and 1000 s/mm^2^). All images were in DICOM format before importing them into MATLABR2020b, as shown in Figure 1. Texture features were extracted at a resolution of 320 × 320 × 19 voxels, and the intensities within each region of interest (ROI) were normalised to a range of 0–1.

### 2.3. Image Processing and Analysis of Textural Feature Extraction

ROIs were segmented from both T2WI and ADC images. For consistency between the two segmented images, all ROIs were carefully mapped out with the same criteria and were visually validated by an expert radiologist with more than 10 years of experience in uro-radiology. This was followed by the extraction of quantitative texture features. The regions of interest were matched to histopathology using 3-D printed moulds based on a previous publication [37]. The derived T2WI and ADC texture features were derived from the histogram and GLCM (Haralick texture features) [38,39] (Supplementary Tables S1 and S2). Second-order statistics features include the statistical inter-relationships between neighbouring voxels [40]. They represent the spatial arrangement of the voxel intensities and hence of intra-lesion heterogeneity. Such features can be derived from the GLCM, quantifying the incidence of voxels with the same intensities at a predetermined distance along a fixed direction [40,41]. This mathematical morphology or phenotype is then correlated with genetic phenotyping of cancers [42].

### 2.4. Histopathology Protocol

The radical prostatectomy specimens from the 15 patients included in this study were labelled, weighed, and formalin fixed. Subsequently, the radical prostatectomy specimens were sliced in patient-specific moulds to assist in correlating and adjusting images with histology. The moulds were fabricated using a 3D printer, as described in a previous study [37]. In each patient, corresponding tumour lesions were assigned a pathologic Gleason grade score, which was reported by an experienced uro-pathologist who was blinded to patients’ imaging reports and subsequently reclassified into three groups using the International Society of Urological Pathology (ISUP) grading system [43,44,45] (Supplementary Table S3).

### 2.5. Profiling of Gene Expression

Formalin-fixed, paraffin-embedded (FFPE) human prostate tissue sections of human prostates were provided to BioClavis (Glasgow, UK) for targeted RNA expression profiling using the TempO-Seq assays (https://www.bioclavis.co.uk, accessed on 9 June 2021) [46,47]. Samples from two different locations of interest, normal prostate tissues and malignant tissues, were identified in H&E-stained slices, with the help of an expert uro-pathologist. The normal and tumour regions of interest were annotated on adjacent unstained tissue sections, which were sent to BioClavis. A 10 mm^2^ excision of each tissue region of interest was processed, using the Human Whole Transcriptome v2.0 TempO-Seq panel with standard attenuators. Internal processing control RNA and no-sample negative controls were run as replicates on each assay plate, together with the samples, to ensure quality metrics passed on a plate-wise level.

### 2.6. TempO-Seq Assay Protocol Summary

Sequencing libraries for targeted panels were generated as described briefly here and depicted in Supplementary Figures S2 and S3. Each Detector Oligo in TempO-Seq was made up of a universal primer-binding site (i.e., one that is the same for all targeted genes) and a sequence complementary to an mRNA target. They immediately anneal next to one another on the specific RNA template, allowing for ligation. Ligated detector oligos were PCR-amplified using a primer set (singleplex PCR reaction, with a single primer pair for each sample) that introduced the adaptors needed for sequencing, as well as a sample-specific barcode. The barcode sequences flanked the target sequence and were inserted appropriately into the standard Illumina adaptors to permit standard dual-index sequencing of the barcodes and deconvolution of sample-specific reads from the sequencing data using the standard Illumina software. All PCR-amplified and barcoded samples were pooled into a single library for sequencing on an Illumina HiSeq 2500 High Output v4 flow cell. Sequencing reads were de-multiplexed using BCL2FASTQ software (Illumina, San Diego, CA, USA) for each sample, using the barcodes, to give a FASTQ file for each. FASTQ files were aligned to the Human Whole Transcriptome v2.0 panel, which consists of 22,537 probes, using STAR in the TempO-SeqR software package [48] (BioSpyder Technologies, Carlsbad, CA, USA), with up to 2 mismatches allowed in the 50-nucleotide target sequence (https://www.bioclavis.co.uk/workflow, accessed on 9 June 2021) [49].

### 2.7. Statistical Analysis

Differential expression was determined by applying the R package DESeq2 to the raw count matrix for the hypoxia gene subset and performing a pairwise comparison between factors (e.g., cancer vs. normal, high vs. low Gleason Score) to obtain the log2fold-change and adjusted *p*-value of the difference in expression levels [50]. Focusing on the differentially expressed hypoxia-related genes of interest, the differential expression studies between cancer and normal tissue were performed after first segregating them by the Gleason score. An exploratory analysis of the differentially expressed hypoxic genes was performed on cBioportal (https://www.cbioportal.org, accessed on 9 June 2021) to further validate their gene output [34]. The genes of interest were searched in the cBiportal gene bank, and an inter-analysis was performed. Correlation analysis for mutation comparisons and mRNA expression vs. mutation type analysis, co-expression analysis using Pearson’s coefficients calculated against each gene expression profile, survival analysis using Kaplan–Meier estimators and plots for overall survival, and mutual exclusivity analysis using the Fisher exact test were conducted in cBiportal. The three differentially expressed hypoxia-related genes were further tested with radiomic texture features using a nonparametric approach of the Games Howell test.

## 3. Results

The workflow for the study is indicated in Figure 1, while Figure 2 illustrates the inclusion and exclusion criteria applied in this study.

Basic demographic characteristics of patients are shown in Table 1.

TempO-Seq-based RNA profiling data of FFPE tissue sections of radical prostatectomy specimens showed significant differential expression of hypoxia-related genes (ANGPLT4, P4HA1, and VEGFA) in contrast to that in normal prostate tissue (Table 2).

**Figure 3:**
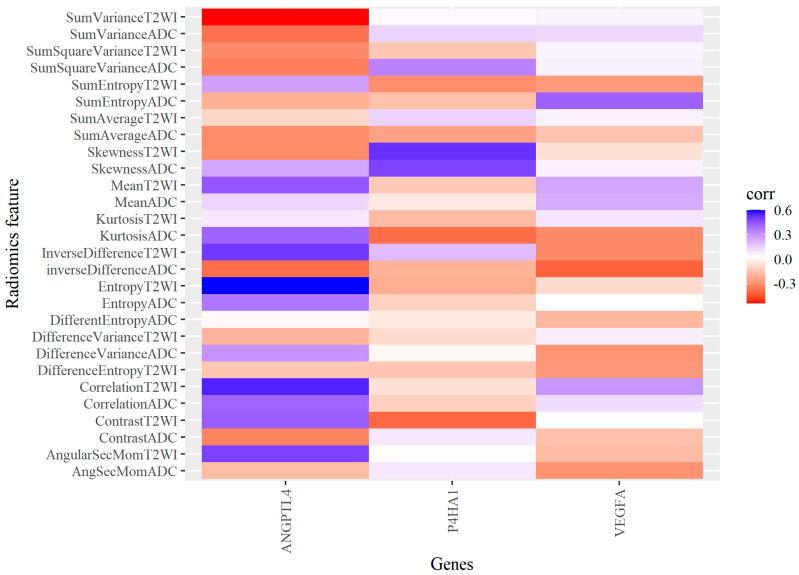
Correlation heatmap showing the three differentially expressed hypoxia-related genes with radiomic texture features.

Figure 3 is a correlation heatmap matrix between RNA-based hypoxia-related gene expression and textural features of pre-biopsy bpMRI in localised prostate cancer. A positive correlation indicates that both variables move in the same direction. A higher degree of textural features indicates higher expression of hypoxia-related genes.

Table 3 shows a significant correlation between the radiomics histogram and GLCM texture features (Sum Variance T2WI, Skewness T2WI, Entropy T2WI, Skewness ADC). For all correlations, Sum Variance T2WI and Entropy T2WI vs. ANGPLT4 showed the highest positive significant correlation (r = 0.600, *p* = 0.020 respectively).

All prostate cancer studies from TCGA using the filter were chosen as a data source for the combination of high-quality genomic and associated clinical data characteristics of queried hypoxic gene datasets in general and the large sample size of the available datasets. The gene query of 9278 samples from 8988 patients reported in 23 studies showed a fraction of the matrix had hypoxia-related genes (ANGPLTL4, P4HA1, and VEGFA). These were altered in 236 (3%) of the reported samples (ratio % distribution 0.5%:3%:0.9% for ANGPLTL4, P4HA1, and VEGFA, respectively, as shown in Supplementary Figure S1). The most common genetic alteration was amplification (copy number alteration) and deep deletion. The analysis of overexpressed hypoxia-related genes was based on three pairs of tests among the three tracks for mutual exclusivity and co-occurrence, as shown in Table 4.

**Figure 4:**
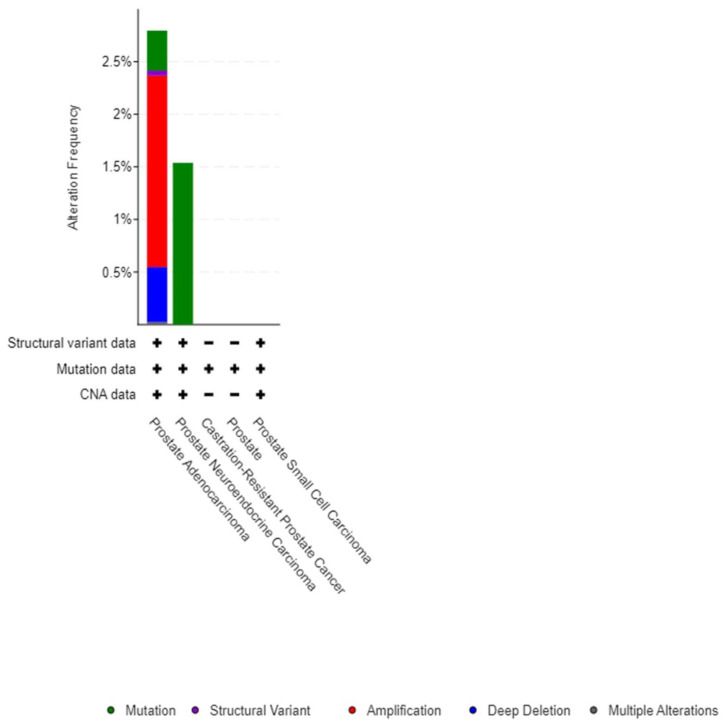
Cancer type summary.

It is apparent that all the three pairs had positive odds ratio values, suggesting that alterations in these genes co-occur in the same samples. This indicates that the three pairs have a significant association with a q-value of <0.05 for each, in addition to having a tendency to co-occur. Cancer type details of overexpressed genes indicated that ANGPLT4, P4HA1, and VEGFA were mostly mutated in prostate adenocarcinoma and prostate neuroendocrine carcinoma (Figure 4).

**Figure 5:**
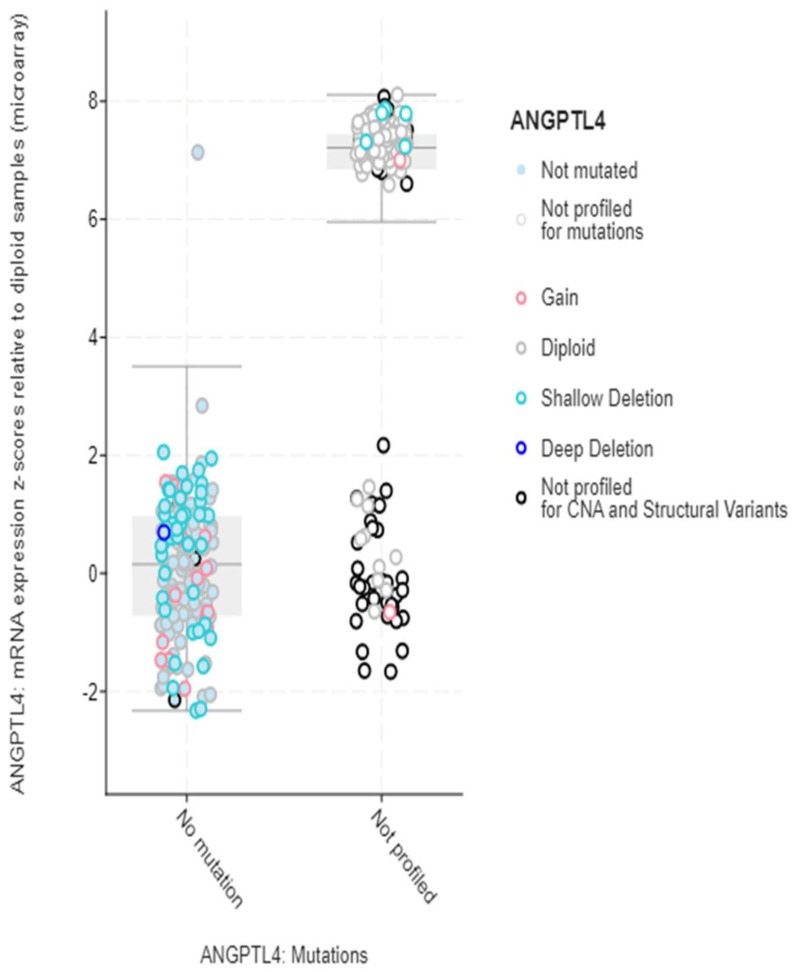
mRNA expression vs. ANGPTL4.

**Figure 6:**
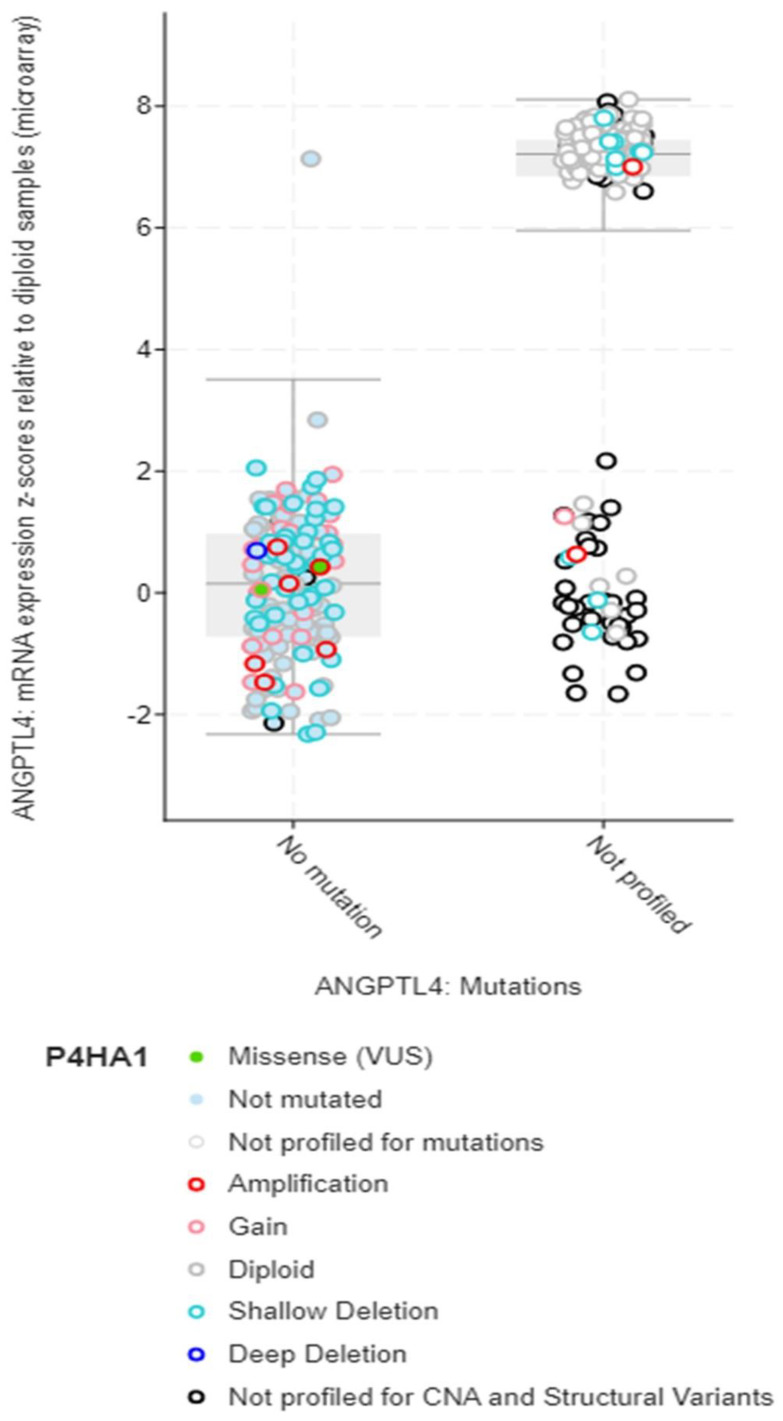
mRNA expression vs. P4HA1.

**Figure 7:**
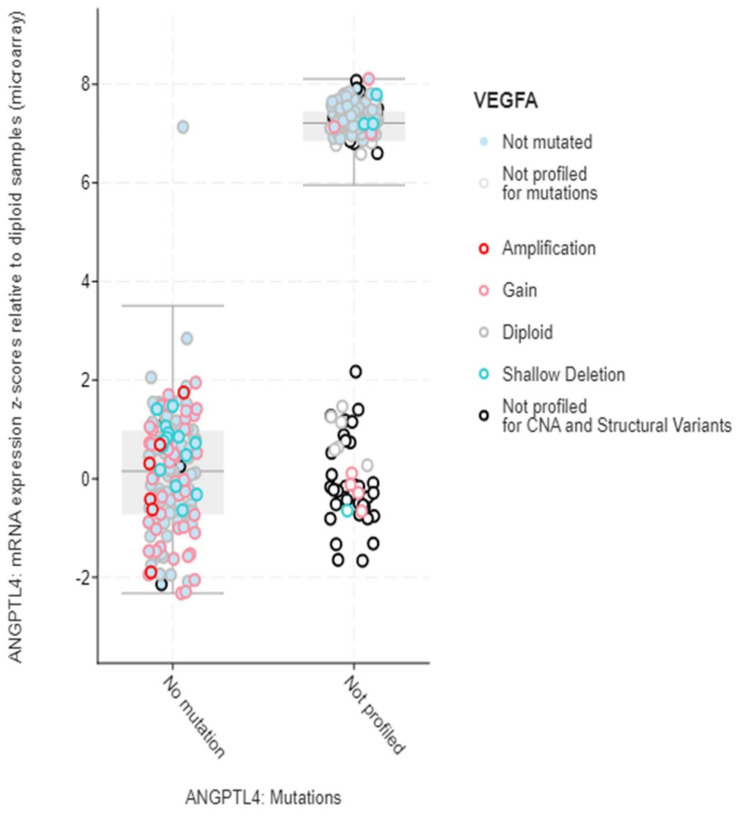
mRNA expression vs. VEGFA.

Structural variant data for the alteration frequency indicated primarily mutation and amplification. The mRNA expression vs. mutation type for ANGPLT4, P4HA1, and VEGFA mRNA expression z-scores relative to diploid samples (microarray) was plotted (Figure 5, Figure 6 and Figure 7).

**Figure 8:**
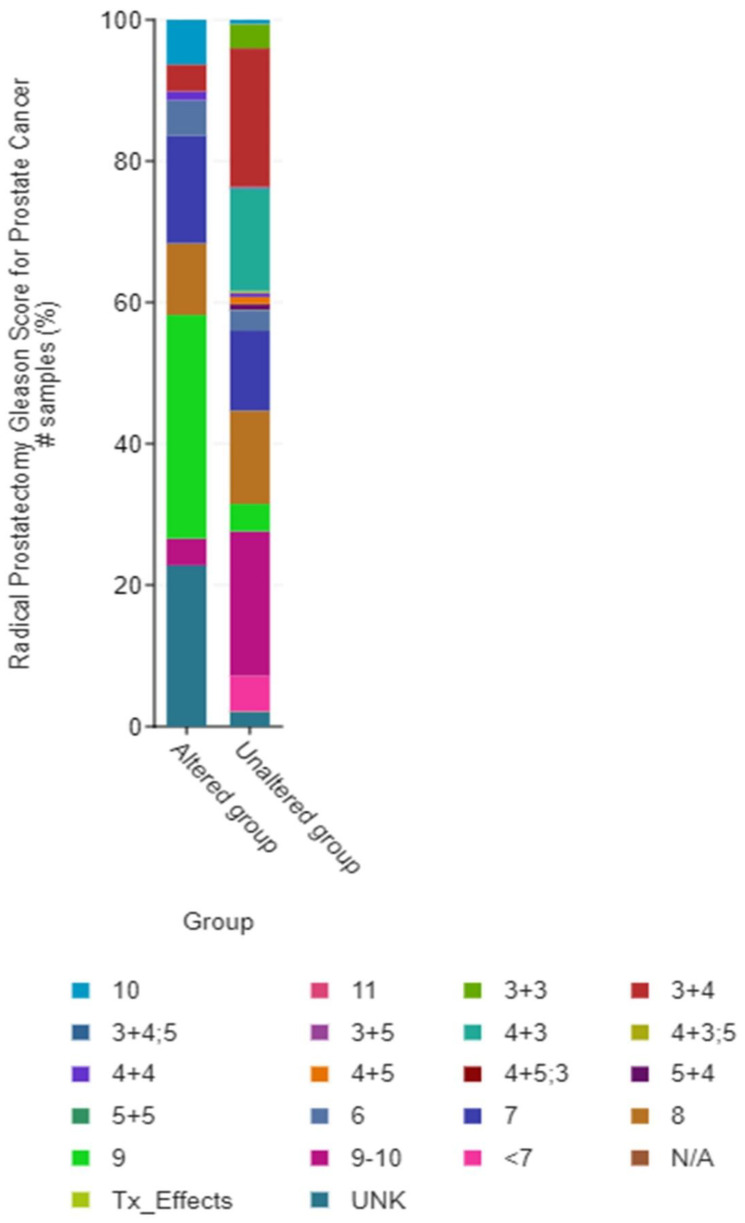
The contribution. Graph of altered and unaltered group.

**Figure 9:**
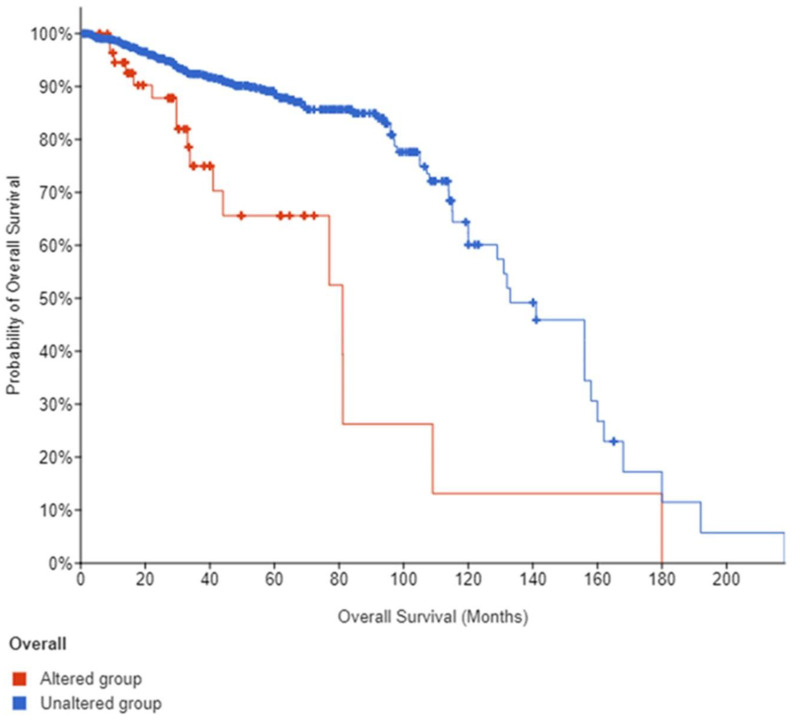
Overall survival data.

Out of the total number of studies that reported ANGPLT4, P4HA1, and VEGFA, 59 samples reported them to be altered, while 1417 reported unaltered genes. The ratio of the altered group to unaltered group for the number of structural variant mutations (number of events) was 17:136. Data showed that the altered group vs. unaltered group had a median survival of 81.11 vs. 133 months, respectively (Figure 8 and Figure 9).

## 4. Discussion

The present study of radiogenomics in localised PCa is both exploratory and hypothesis-driven. Multiple studies have demonstrated the diagnostic potential of metabolomics for prostate cancer and have demonstrated the diagnostic potential of metabolomics and exosomes for the differential diagnosis of prostate cancer [51,52,53,54]. However, this study sought a priority of a pure genomics approach and assessed the correlation between quantitative texture radiomic features of biparametric MRI and hypoxia-related gene expression in men with localised prostate cancer. In the exploratory phase, we report a significant correlation between textural feature analyses of pre-biopsy bpMRI and hypoxia-related gene expression in localised PCa. In the hypothesis-driven section, we report significant differences in survival among patients who had these genes altered (amplification or mutation).

In a recent systematic review [17,55] radiomics features performed very well in making a distinction between Gleason grades, a surrogate marker for PCa aggressivity. The present study furthers this knowledge by exploring the correlation between radio phenotype and genotype, focusing on hypoxia-related changes. Our approach to radiogenomics targeting hypoxia-related genetic changes is similar to that reported by Sun et al. [31] with some key differences. Firstly, we selected men with an equitable distribution of Gleason scores amongst the cohort in contrast to only Gleason score 7 disease. Secondly, pre-biopsy MRI was used for radiomics feature analysis in contrast to post-biopsy scans; thirdly, patient-specific 3-D printed moulds were used to orient histology with the imaging data. Fourthly, we tested the hypothesis of the survival impact of hypoxia-related gene expression using TCGA dataset. Despite obvious differences in the number of genes between the present study and the one reported by Sun et al., amongst the top three differentially expressed genes in the present study, two (VEGFA and P4HA1) were found to be common in both studies [31]. The present study used a different but novel tempO-sequence method to obtain RNA sequencing data in combination with a fold-change level of ≥2, the reason for reporting only three main significant hypoxia-related genes. Most stringent criteria (intersection of *p* ≤ 0.05 and ≥2 fold-changes) in our study are known to reduce gene expression data [56].

Imaging of hypoxia in prostate cancer using a radiogenomics approach has already become an attractive option for avoiding rectal and urinary bladder toxicity in men opting for hypofractionated external beam radiotherapy for localised PCa [57]. Tumour hypoxia has been established as a key biological phenomenon that determines the accumulation and propagation of cancer stem cells and has been implicated in increasing risks of metastases, as well as reducing the effectiveness of radical treatment, including surgery. Hypoxia also affects chemotherapy and chemo-radiation, key interventions in the cancer management of PCa. The goal of the study was to identify and apply a radiogenomics approach to provide insight into the development, heterogeneity, and occurrence of tumours using radiomic and genomic data. The present study showed a significant correlation between the expression of hypoxia-related genes and heterogeneity seen in texture analysis of patients with localised PCa.

Current evidence suggests that the grade of a tumour is directly related to genomic instability, heterogeneity, and composition of the microenvironment, varying for different types of cancers and even among different patients having the same tumour histotype. Our results identified several texture features that were correlated with hypoxia-related gene expression. In the correlation analysis, five bpMRI (T2WI & ADC) features (Difference-entropy T2WI, Skewness ADC, Entropy T2WI, Sum Variance T2WI, and Skewness T2WI) correlated with hypoxia-related gene expression with varying magnitudes.

Previous research has shown that Difference-entropy, which is a measure of the randomness/variability in pixel intensity value differences between neighbours, and entropy, which is a measure of randomness in imaging values, are both promising quantitative imaging biomarkers for characterising cancer phenotypes in imaging and contribute to the assessment of intra- and inter-tumour heterogeneity using a radiomic approach [58,59,60,61]. Skewness measures the asymmetry in the distribution of its pixels or voxels. This statistical parameter quantifies how far the distribution of pixel/voxel values depart from a symmetrical, bell-shaped distribution (i.e., a normal distribution). An image with a positive skewness value may depict a region where there is a higher density or concentration of a specific tissue type, whereas an image with a negative skewness value may depict a region where there is a lower density or concentration of the same tissue type [62,63]. Sum Variance involves adding up all the pixel intensities in a region of interest (ROI) and dividing the result by the variance of those same ROI’s pixel intensities. The obtained value is then used as a radiomic feature to reveal the heterogeneity or texture of the ROI. According to the Sum Variance approach, regions with high heterogeneity will have a greater sum of pixel intensities and a lower variance, whereas regions with low heterogeneity would have a lower sum of pixel intensities and a larger variance [64]. Studies have shown that higher Sum Variance values are associated with more aggressive and diverse prostate tumours, as well as a higher risk of cancer recurrence after therapy [65,66,67,68,69]. Gene expression is also subject to genetic changes and intra-tumour heterogeneity [70,71].

This study established the differential expression of three key hypoxia genes, ANGPTL4, P4HA1, and VEGFA, which were correlated with several radiomic texture features, mainly focusing on tumour heterogeneity. The genes expressed in prostate cancer in this study are reported to have a correlation with extracellular matrix organisation, metabolic pathways, and signalling pathways, which provides a plausible link between genomic-driven biology and bpMRI texture features. Angiopoitin-like-4 gene expression (ANGPTL4) showed a significant positive correlation with the Entropy T2WI and Sum-variance T2WI (r = 0.600, *p*-value 0.020). Furthermore, the ANGPTL4 gene was associated with high-Gleason score disease (L2FC = 6.07, *p* = 0.050). The pathway description indicated the ANGPTL4 gene is implicated in the PPAR signalling pathway and modulates lipid metabolism via an oligomerisation process.

ANGPLT4 inhibition was reported to be associated with a significant decrease in neovascularisation in vitro and encodes G protein-coupled receptor (vGPCR)-mediated tumorigenesis in vivo [72]. Though various reports have focused on various forms of cancer, this study lays credence to the discovery of the possible implication of ANGPTL4 in prostate cancer and its key association with a high Gleason score and radiomic features.

P4HA1 expression showed a significant correlation with Skewness T2WI (r = 0.524, *p*-value 0.024). P4HA1 encodes part of the collagen biosynthesis and modifying enzyme; collagen is considered an important makeup of cellular structures. Various reports have linked the P4HA1 gene to cancer proliferation and hypoxic up-regulation [2,3,4,73], and overexpression has been reported in gliomas and HNSCC; expression has also been linked to tumour micro-vessel density [5]. The gene pathway association between these overexpressed hypoxia genes would be via the PVT1/PRC2/STAT3 pathway and the ERK/HIF-1α/p70S6K cascade, which signals tumour-induced angiogenesis [5].

In our observations, the VEGFA gene was significantly associated with a medium Gleason score (L2FC = −2.17, *p* = 0.006) and P4HA1 (L2FC = 2.16, *p* = 0.047). The VEGFA gene has been implicated in types of cancer, including prostate cancer, as a validation gene query based on a cBioprtal 4% alteration in 4946 sample repositories suggests. The pathway description indicated the VEGFA gene is implicated in various signalling pathways in cancer, a main inducer of blood vessel growth and an important growth factor for epithelial cells [72].

The findings of the study also lay support to already published data, showing poor survival in men with a hypoxia-related genetic PCa signature, mapping phenotypes of these cancers based on imaging using radiomic features, which informs further research in this area. Knowledge gained through this and future research would provide evidence to support the need for the consideration of personalised treatments and the promotion of non-invasive methods by creating imaging biomarkers, which will play key roles in monitoring tumour heterogeneity.

There are various limitations to this study. Firstly, due to the small patient cohort size, the resulting output was insufficient to develop a predictive model. However, this study substantiates a proof-of-concept and provides empirical evidence for future research with larger samples; secondly, pathway analysis with gene set enrichment analysis and subject permutation could not be performed as it would require a complete transcriptome rather than just a subset of genes; and thirdly, the observed findings show relatively few lesions in the transitional zone compared to those in the peripheral zone, which in contrast to equality distribution, may contribute to confounding bias, as tumour stratification was not considered in texture feature extraction. In the future, radiomics could incorporate other imaging modalities, such as prostate-specific membrane antigen positron emission tomography (PSMA-PET), and explore their correlation with genomics.

In conclusion, this study revealed that there is a noteworthy connection between various hypoxia-related genes that are differentially expressed and the quantitative radiomic texture features extracted from pre-biopsy bpMRI in prostate cancer cases that are clinically localised. The cBiopotal data further confirmed that changes in these hypoxia-related genes can lead to poor survival outcomes in prostate cancer.

## Figures and Tables

**Table 1:** Patient characteristics.

Characteristics Median	PCa (n)	(Median-IQR)
Age (years)	15	66–13
Size (mL)	15	51.5–22
Prostate location		
Transitional zone	4	
Peripheral zone	11	
PSA (ng/mL)		
PSA 4–20	15	10.8–4.2

**Table 2:** A single significant result was obtained for each category after first grouping by Gleason score and then attempting the differential expression analysis between cancer and normal tissue.

Genes	Gleason Risk Category	BaseMean	log2Fold-Change	*p*-Value
P4HA1	low	84.1	2.16	0.047
VEGFA	Intermediate	902.9	−2.17	0.006
ANGPLT4	high	6.07	6.07	0.050

**Table 3:** Corelation between radiomic histogram and GLCM texture features with three differentially expressed hypoxia genes in prostate cancer (*p*-value < 0.05).

Radiomic Features	ANGPLT4		P4HA1		VEGFA	
	r	*p*-Value	r	*p*-Value	r	*p*-Value
Sum Variance T2WI	0.600	0.020	0.045	0.048	−0.151	0.042
Skewness T2WI	−0.312	0.034	0.524	0.024	−0.061	0.047
Entropy T2WI	0.600	0.020	0.043	0.048	−0.079	0.046
Skewness ADC	0.247	0.038	0.493	0.025	0.060	0.047

**Table 4:** Mutual exclusivity and co-occurrence of overexpressed hypoxia genes (cBioportal).

Panel A	Panel B	Neither	A Not B	B Not A	Both	Log2 Odds Ratio	*p*-Value	q-Value	Tendency
ANGPLT4	VEGFA	4373	15	55	8	>3	<0.001	<0.001	Co-occurrence
P4HA1	VEGFA	4246	142	55	8	2.121	0.001	0.001	Co-occurrence
ANGPLT4	P4HA1	4282	19	146	4	2.826	0.007	0.001	Co-occurrence

## Data Availability

URL: http://helpdesk.ebi.ac.uk/Ticket/Display.html?id=637816, accessed on 27 March 2023 submission with accession number E-MTAB-12593. Release date: 2023-01-29. You will be able to see it only by logging in or by accessing it through this link: https://www.ebi.ac.uk/biostudies/arrayexpress/studies/E-MTAB-12593?key=7ee20e91-4252-436a-9c8f-cb7613e23309, accessed on 27 March 2023.

## Supplementary Materials

The following supporting information can be downloaded at: https://www.mdpi.com/article/10.3390/jcm12072605/s1. Figure S1: Oncoprint Gene Query of Overexpressed hypoxia genes to cbioportal prostate cancer studies; Figure S2: Lysate preparation from FFPE prostate sample slides; Figure S3: Biochemistry of TempO-Seq; Table S1: Summary for radiomic features; Table S2: Description of Histogram and Grey-level co-occurrence Metrix (GLCM) features computed within the region of interest (ROI) of prostate tumour; Table S3: Gleason grading system.
