# Supplementary material for: Radiogenomics Reveals Correlation between Quantitative Texture Radiomic Features of Biparametric MRI and Hypoxia-Related Gene Expression in Men with Localised Prostate Cancer
Source: J Clin Med. 2023 Mar 30;12(7):2605. doi: 10.3390/jcm12072605 (PMC10095552; doi:10.3390/jcm12072605)
Supplement: Supplementary file 1 [file jcm-12-02605-s001.zip › SUPPLEMENTARY TABLES for article 2 02-03-23.pdf]

Supplementary Tables

- **Supplementary Table S1.** Summary for radiomic features.

	Variable	Mean	Median	SD	Min	Max
1	AngularSecMomT2WI	0.4	0.3	0.14	0.2	0.6
2	ContrastT2WI	69.1	53	52.644	20	188
3	CorrelationT2WI	0.6	0.6	0.118	0.3	0.8
4	SumSquareVarianceT2WI	96.3	95	23.427	50	146
5	InverseDifferenceT2WI	0.3	0.2	0.206	0.1	0.8
6	SumAverageT2WI	59.6	64	9.156	30	65
7	SumVarianceT2WI	41.4	36	13.767	20	68
8	SumEntropyT2WI	3.9	3.6	0.689	3.1	5.1
9	EntropyT2WI	2.8	2.6	1.256	1.1	5
10	DifferenceVarianceT2WI	40.6	41	7.189	20	52
11	DifferenceEntropyT2WI	3.2	3.1	1.441	1.2	6.3
12	AngSecMomADC	0.5	0.4	0.449	0.3	2.1
13	ContrastADC	44.5	48	13.999	18	66
14	CorrelationADC	0.5	0.5	0.264	0.1	0.9
15	SumSquareVarianceADC	70.3	52	23.578	50	102
16	inverseDifferenceADC	0.5	0.5	0.206	0.1	0.9
17	SumAverageADC	44.5	36	27.689	9	93
18	SumVarianceADC	36.7	36	5.64	27	47
19	SumEntropyADC	3.2	2.8	0.916	1.8	4.5
20	EntropyADC	1.4	1.3	0.342	1	2.1
21	DifferenceVarianceADC	27.5	27	9.149	11	43
22	DifferentEntropyADC	1.5	1.2	0.897	1	4.6
23	MeanT2WI	33.4	34	8.399	16	43
24	MeanADC	42.7	47	11.412	19	58
25	SkewnessT2WI	0.4	0.4	0.389	0.1	1.4
26	SkewnessADC	0.6	0.5	0.511	0.1	1.8
27	KurtosisT2WI	1.1	0.4	1.379	0.1	3.7
28	KurtosisADC	0.8	0.6	0.829	0.1	2.7

Supplementary Table S2: Description of Histogram and Grey-level co-occurrence Metrix (GLCM) features computed within the region of interest (ROI) of prostate tumour**.**

**Texture Features**	**Description**
**Histogram (first order features)**	**They are derived from first order statistics and provides information related to grey level distribution of ROI.**
Mean	Average grey level intensity
Skewness	Measures the spread distribution of values about the average value.
Kurtosis	Measures the peakedness of distribution of values in image.
**Grey-level co-occurrence matrix (GLCM)**	**(2nd order statistic Characterizing spatial relationship between intensity values within ROIs).**
Angular Second moment	Measures the textural uniformity that is pixel pair recurrences. It detects disorder in textures
Contrast	Measures local intensity variations
Correlation	Explains the linear dependency of Grey level values to their respective voxels in the GLCM
Sum Square Variance	Measures the distribution of neighbouring intensity level pairs about the average of intensity level in the GLCM
Inverse Difference	Measures of the local homogeneity of an image
Sum Average	Measures the relationship between occurrences of pairs with lower intensity values and occurrences of pairs with higher intensity values
Sum Variance	Explains the weights elements that differ from the average value of the GLCM.
Entropy	Explains the randomness in the GLCM
Sum Entropy	Explains the sum of neighbourhood intensity value differences.
Difference Variance	Measure of heterogeneity that places higher weights on differing intensity level pairs that deviate more from the mean.
Difference Entropy	Measure of the randomness/variability in neighbourhood intensity value differences.

Supplementary Table S3: Gleason grading system.

**Gleason (n)**	**Gleason score**	**Risk Group**
Grade Group 1 (5)	≤ 6	Low
Grade Group 2 (5)<br>Grade Group 3 (0)	3+4=7<br>4+3=7	Medium or Intermediate
Grade Group 4 (3)<br>Grade Group 5 (2)	4+4=8; 3+5=8 ; 5+3=8 or 9	High
