# Supplementary figures and images for: Radiogenomics Reveals Correlation between Quantitative Texture Radiomic Features of Biparametric MRI and Hypoxia-Related Gene Expression in Men with Localised Prostate Cancer
Source: J Clin Med. 2023 Mar 30;12(7):2605. doi: 10.3390/jcm12072605 (PMC10095552; doi:10.3390/jcm12072605)

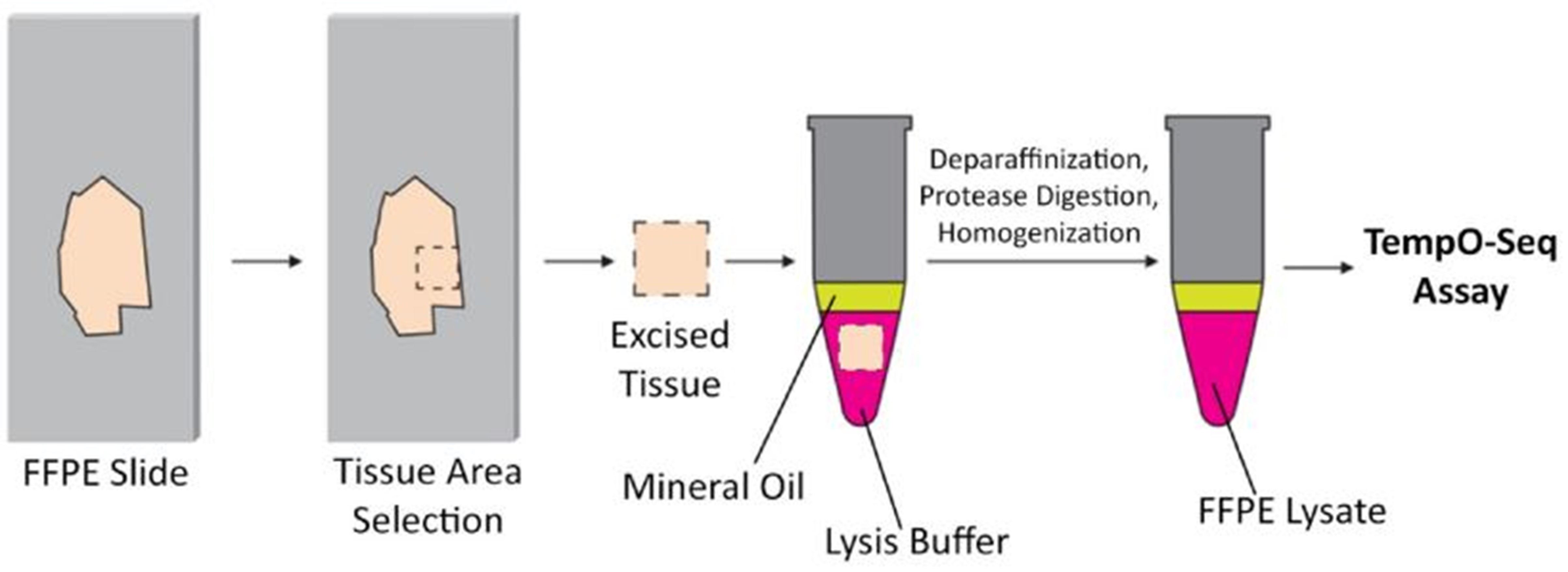

Supplement: Supplementary file 1 [file jcm-12-02605-s001.zip › [Supplementary Figure S2] Lysate preparation from FFPE prostate sample slides.JPG]

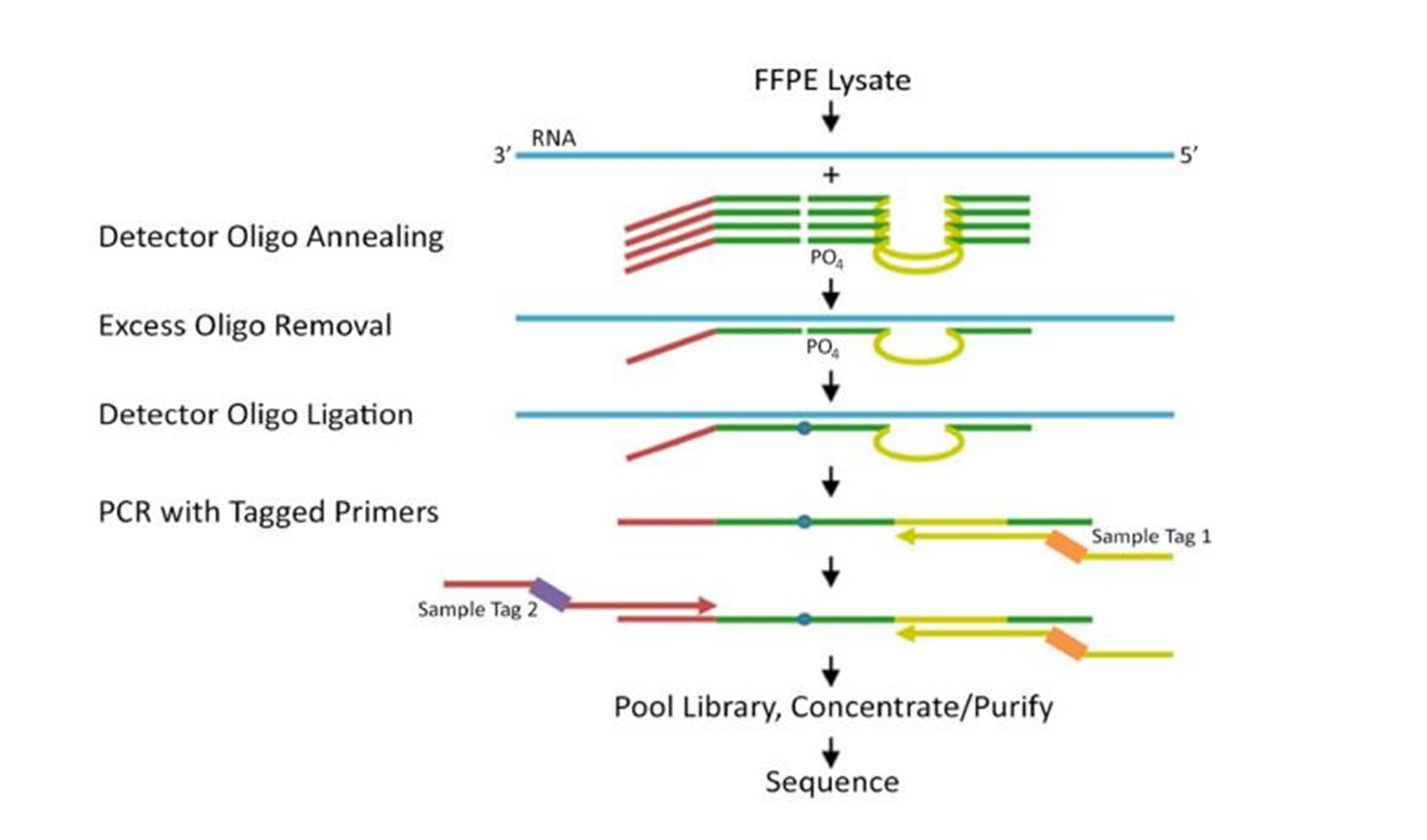

Supplement: Supplementary file 1 [file jcm-12-02605-s001.zip › [Supplementary Figure S3] Biochemistry of TempO-Seq.JPG]
